# Imaging in staging, treatment planning, and monitoring of hepatocellular carcinoma for local and locoregional therapies: consensus recommendations from EORTC and ESGAR

**DOI:** 10.1007/s00330-025-11699-7

**Published:** 2025-05-23

**Authors:** Osman Öcal, Christoph Johannes Zech, Maria Antonietta Bali, David Pasquier, Felix Mottaghy, Ingo Einspieler, Nikolaos Kartalis, Irene Bargellini, Roberto Iezzi, Timm Denecke, Wolfgang Gerhard Kunz, Bernhard Gebauer, Henning Wege, Roberto Cannella, Daniela Elena Oprea-Lager, Arndt Vogel, Bruno Sangro, Max Seidensticker

**Affiliations:** 1 Department of Diagnostic and Interventional Radiology, Heidelberg University Hospital, Heidelberg, Germany; 2 Imaging Group, European Organisation of Research and Treatment in Cancer (EORTC), Brussels, Belgium; 3 Radiology and Nuclear Medicine, University Hospital Basel, University of Basel, Basel, Switzerland; 4 European Society of Gastrointestinal and Abdominal Radiology (ESGAR), Vienna, Austria; 5 Radiology Department, Institut Jules Bordet Hôpital Universitaire de Bruxelles, Université Libre de Bruxelles, Brussels, Belgium; 6 Academic Department of Radiation Oncology, Centre Oscar Lambret, Lille, France; 7 CRIStAL, CNRS, Lille University, Lille, France; 8 Department of Radiology and Nuclear Medicine, Maastricht University Medical Center, Maastricht, The Netherlands; 9 Department of Nuclear Medicine, University Hospital RWTH Aachen University, Aachen, Germany; 10 Department of Radiology, University Hospital Regensburg, Regensburg, Germany; 11 Division of Radiology, Department of Clinical Science, Intervention and Technology (CLINTEC), Karolinska Institute, Stockholm, Sweden; 12 Diagnostic and Interventional Radiology Unit, Candiolo Cancer Institute, FPO-IRCCS, Candiolo, Italy; 13 Dipartimento di Diagnostica per Immagini e Radioterapia Oncologica, Fondazione Policlinico Universitario A. Gemelli IRCCS, UOC di Radiologia d’Urgenza e Interventistica, Roma, Italia; 14 Institute of Radiology, Catholic University, Rome, Italy; 15 Department of Diagnostic and Interventional Radiology, University of Leipzig, Leipzig, Germany; 16 Department of Radiology, University Hospital, LMU Munich, Munich, Germany; 17 Department of Radiology, Charité—University Medicine Berlin, Berlin, Germany; 18 Cancer Center Esslingen, Klinikum Esslingen, Esslingen, Germany; 19 Section of Radiology, Department of Biomedicine, Neuroscience and Advanced Diagnostics (BiND), University of Palermo, Palermo, Italy; 20 Department of Medical Imaging, Radboud University Medical Center, Nijmegen, The Netherlands; 21 Division of Gastroenterology and Hepatology, Toronto General Hospital, Toronto, ON, Canada; Medical Oncology, Princess Margaret Cancer Centre, Toronto, ON Canada; 22 Department of Gastroenterology, Hepatology, Infectious Diseases and Endocrinology, Hannover Medical School, Hannover, Germany; 23 Liver Unit and HPB Oncology Area, Clínica Universidad de Navarra and CIBEREHD, Pamplona, Spain; 24 Sapienza University of Rome, Rome, Italy; 25 La Fe Health Research Institute (IIS La Fe), Valencia, Spain; 26 Bern University Hospital (Inselspital), Bern, Switzerland; 27 Niels-Stensen-Kliniken Marienhospital, Osnabrück, Germany; 28 Amsterdam UMC, Amsterdam, The Netherlands; 29 Hacettepe University, Ankara, Turkey; 30 Ospedale del Mare, Naples, Italy; 31 Maastricht University Medical Center, Maastricht, The Netherlands; 32 UZ Leuven, Leuven, Belgium; 33 University and Hospital Trust, Verona, Italy; 34 Hampshire Hospitals NHS Foundation Trust, Basingstoke, UK; 35 University Hospital of Magdeburg, Magdeburg, Germany; 36 Leiden University Medical Center, Leiden, The Netherlands; 37 Ramón y Cajal University Hospital, Madrid, Spain; 38 University Hospitals Bristol, Bristol, UK; 39 Giresun University Hospital, Giresun, Turkey; 40 San Giovanni Hospital, Bellinzona, Switzerland; 41 University Hospital Regensburg, Regensburg, Germany; 42 Charité Universitätsmedizin Berlin, Berlin, Germany; 43 Department of Biomedical Sciences, Humanitas University, Pieve Emanuele, Milan, Italy; 44 Humanitas Cancer Center, IRCCS Humanitas Research Hospital, Rozzano, Milan, Italy; 45 Klinikum Würzburg Mitte, Würzburg, Germany

**Keywords:** Hepatocellular carcinoma, Imaging, Ablation, TACE, SBRT

## Abstract

**Background:**

Periinterventional imaging of patients with hepatocellular carcinoma (HCC) during local and locoregional therapies plays a crucial role in clinical outcome by guiding treatment allocation, planning, and application. However, there is a considerable variety in clinical routine in terms of timing, modality, and imaging protocols. This study aimed to guide the standardization of the imaging procedures for patients with HCC by conducting a Delphi consensus-finding survey.

**Methods:**

A multidisciplinary, multinational survey was conducted to standardize the imaging of patients with HCC using the Delphi method.

**Results:**

Under the guidance of the European Organisation for Research and Treatment of Cancer (EORTC) Imaging and Gastrointestinal Tract Cancer Groups and the European Society of Gastrointestinal and Abdominal Radiology (ESGAR), the recommendations for imaging before, during, and after thermal ablation, transarterial chemoembolization, radioembolization, and stereotactic body radiation therapy were established.

**Conclusion:**

This consensus protocol provides a foundational guide for imaging in the daily clinical management of HCC patients, as well as for prospective studies assessing local and locoregional therapies.

**Key Points:**

***Question*** *There are clear recommendations for the respective therapies/disease stages in HCC, but only to a limited extent for all-around imaging of local therapies*.

***Findings*** *This study conveyed a Delphi consensus-finding survey amongst European experts from multiple medical fields to standardize the periinterventional imaging of HCC patients*.

***Clinical relevance*** *These recommendations can guide both daily clinical practice and prospective trials focused on local and locoregional therapies*.

**Graphical Abstract:**

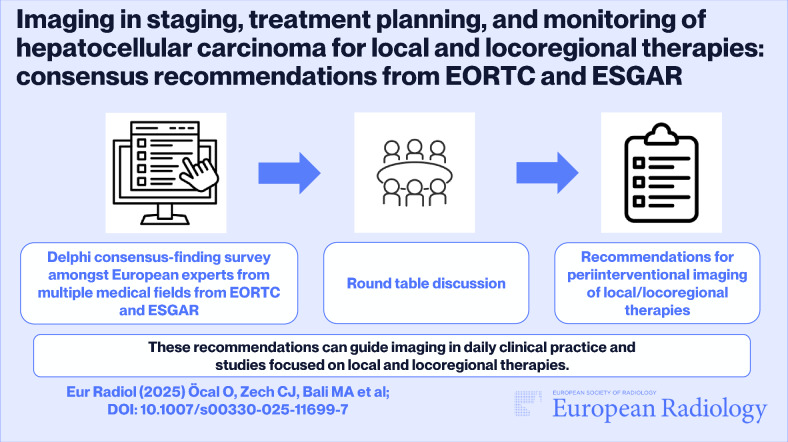

## Introduction

Liver cancer was the seventh most common cancer and the second leading cause of cancer-related mortality in 2020, with hepatocellular carcinoma (HCC) accounting for approximately 90% of the primary liver tumors [1]. Local and locoregional therapies for HCC have been shown to be beneficial in various stages of the disease and are widely utilized as standard of care.

Local ablative therapies offer a minimally invasive alternative to surgical resection. Intra-arterial therapies are the primary locoregional therapy options in selected patients in the intermediate stage of disease (good liver function, good arterial supply), and treatments such as radiation segmentectomy offer an effective alternative for patients with early-stage disease. Trials and series over the past two decades have demonstrated the efficacy and safety of stereotactic body radiation therapy (SBRT) for HCC, leading to its inclusion as an alternative treatment to the standard of care in recent guidelines [2, 3].

Therapy decisions require evaluation of the patient in multidisciplinary tumor boards, considering oncological and medical aspects, as well as the technical feasibility of the local ablative or locoregional therapies.

Imaging is essential not only for staging and treatment planning but also to guide the procedure, monitor therapeutic success, and guide further interventions. Although non-invasive diagnosis based on imaging features has been widely adopted in the past, there is considerable heterogeneity in imaging procedures, including timing, choice of modality, and imaging parameters. Additionally, depending on the locoregional treatment used, these parameters might need to be altered in follow-up imaging. Considering the increasing number of trials evaluating the combination of locoregional therapies with systemic treatments, expert guidance on how best to perform imaging before, during, and after the procedure is needed. This study aimed to standardize the imaging procedures for patients with HCC by conducting a Delphi consensus-finding survey amongst European experts from multiple medical fields focusing on HCC treatment.

## Methods

We conducted a prospective, multistep, modified, non-anonymous Delphi consensus-finding survey. Members of the European Organisation for Research and Treatment of Cancer (EORTC) Imaging Group, the EORTC Gastrointestinal Cancer Group, and the European Society of Gastrointestinal and Abdominal Radiology (ESGAR) were invited to participate in the survey. Two authors have led the process for the EORTC Imaging Group and constructed and edited the questions for each round. Questionnaires were created using Google Forms (https://www.google.com/forms), and access links were shared with participants through the EORTC office.

The first step aimed to collect general information regarding the expertise and local practice of participants in order to obtain a general overview. All questions were multiple choice, but with an option to provide open-ended answers (Supplementary File 1). Considering the range of specialties, the participants were allowed to skip questions. Based on the answers from the first round and the respective literature review performed by the two leading authors, recommendations were formed for the second round of the survey, which accepted only dichotomous, yes and no, answers (Supplementary File 2). Due to the binary nature of the questions, an agreement of 70% was considered a consensus. After the second round, all participants were invited to an online meeting to discuss the results. The results of each question and recommendation were discussed, and the potential reasons for failure to reach consensus were evaluated. Based on the comments in this discussion round, a third round of survey with a single question was performed due to the suggestion of rephrasing the question.

## Results

### Participant/institution characteristics

**Fig. 1:**
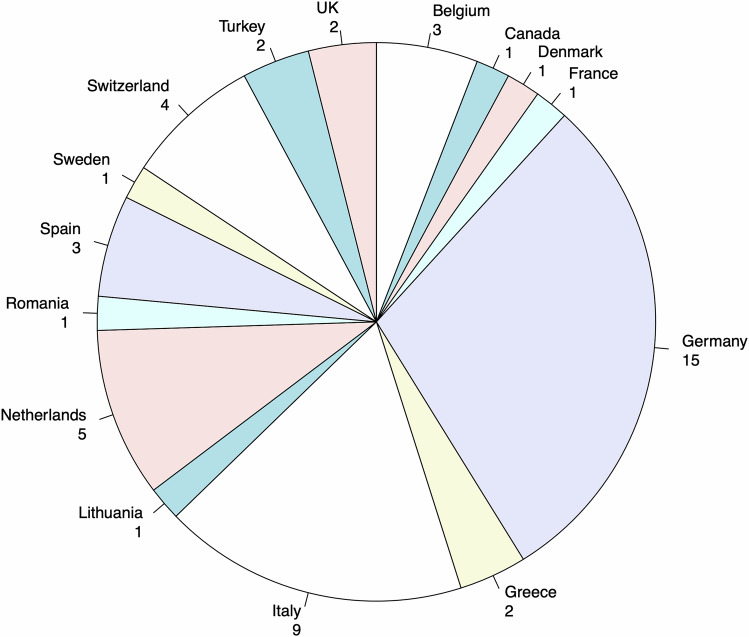
Country of origin of survey participants

**Fig. 2:**
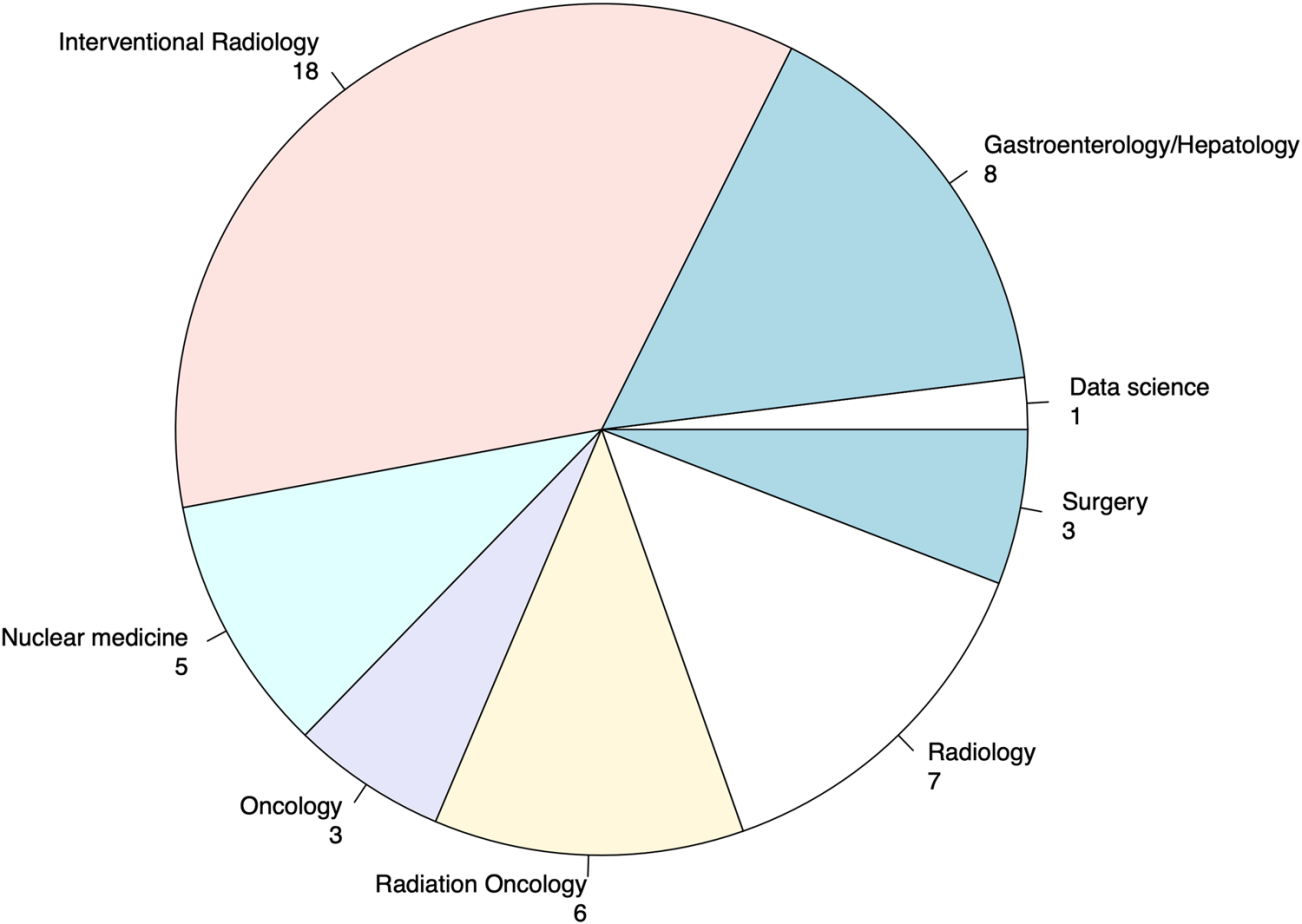
Profession of survey participants

Fifty-one participants from 15 countries participated in the first round of the survey (Fig. 1). All questions from the first survey round are presented in the Supplementary File 1. Thirty-seven participants were working at university hospitals, eleven at tertiary centers, and three at research facilities. Interventional radiologist (*n* = 18) was the most common profession, followed by gastroenterologist/hepatologist (*n* = 8), radiologist (*n* = 7), radiation oncologist (*n* = 6), nuclear medicine physician (*n* = 5), medical oncologist (*n* = 3), surgeon (*n* = 3), and data scientist (*n* = 1) (Fig. 2). Of those 51, 37 participants also completed the second round of the survey.

In more than 50% of the centers, more than 40 local ablation or transarterial chemoembolization (TACE) procedures were performed yearly. The yearly radioembolization procedures were > 20 in 29.5% of centers and < 20 in 40.9%, and yearly SBRT procedures were more than 5 in 40% of centers and < 5 in 26.6% of centers.

### Preferred treatment modalities

The most practiced treatment was ablation (60%) for Barcelona Clinic Liver Cancer (BCLC)-0 patients, ablation (49%), or resection (36%) for BCLC-A patients in the centers of participants. In patients waiting for transplantation, the treatment modality for bridging (treatment of accepted transplant candidates) was TACE + ablation (36%), TACE (31%), ablation (26%), and transarterial radioembolization (TARE, 7.1%). Similarly, these treatments were also used for downstaging, TACE (37%), TACE + ablation (32%), or TARE (20%).

The decision to combine TACE with ablation in patients with BCLC stage 0 or A was driven by lesion size exceeding 3 cm in 31% of cases, challenging tumor locations in 22%, and consistently in 8.9% of cases. Thirty-four percent of participants avoid thermal ablation in lesions with proximity to the liver hilum, 16% in lesions with proximity to the liver hilum or perivascular location, and 16% in lesions with proximity to the liver hilum or perivascular location or subcapsular location. The decision to use TACE was taken using respective criteria (i.e., ABCR score) by 34% of the participants, while 27% avoided TACE in hypovascular lesions. Similarly, 43% use some previously published criteria to decide when not to perform TARE, while 16% have no restrictions for TARE, and 11% for hypovascular lesions. 36% have no restrictions for SBRT, whereas 32% avoid SBRT in lesions with proximity to the liver hilum, and 11% in perivascular locations.

Notably, the majority of participants (87%) are screening patients undergoing systemic therapy for potential locoregional interventions. Given the rising response rates with systemic treatments, this approach will become increasingly crucial in achieving tumor-free status in a growing number of patients, ultimately enhancing long-term survival outcomes.

### Imaging work-up

The majority of participants (63%) use both CT and MRI for imaging work-up of HCC patients, while 33% use only MRI and 3.9% only CT. Gadoxetic acid is used consistently by 71%, occasionally by 11%, and never by 16%. In 60% of the centers, CT protocol for abdomen imaging consists of unenhanced images followed by triphasic (arterial, portal, and venous) dynamic series, whereas 33% perform CT with only triphasic dynamic series, and the rest with only arterial and portal phase series. The imaging delay for venous phase images in abdominal CT is 120 s in 45% of participants´ institutions and 180 s in another 45%. Diffusion-weighted images (DWI) are routinely obtained by the vast majority (96%). 61% of the participants always perform Thorax CT in the imaging work-up, and 27% sometimes. The accepted interval between imaging and locoregional procedure is up to 1 month by 81% of the participants and up to 3 months by 10%. This interval is up to 1 month or three months each, by 48% of the participants, for extrahepatic imaging.

#### Consensus round: imaging work-up

In total, 83.8% of the participants agreed that CT and MRI should be obtained to evaluate the local disease load in the liver, and 89.2% agreed that gadoxetic acid should be used as the contrast agent for the MRI of the liver. Acquisition of DWI in liver MRI was deemed mandatory by 89.2% of the participants. A majority argued that abdominal CT imaging for HCC patients should include arterial, portal, and venous phase images (97.3%). No consensus was reached, with 56.8% of the participants recommending that pre-contrast images should always be obtained for abdominal CT of HCC patients. A majority (77.8%) agreed that the delay for venous phase images should be 180 s for abdominal CT. Most (86.5%) agreed that thorax CT should be obtained in all patients at the baseline. 83.8% of participants agreed that PET-CT studies are not mandatory for imaging work-up in HCC patients. The majority argued that (78.4%) the images should be repeated if liver images are older than one month. However, this interval could be up to 3 months for thorax CT images (78.4%).

### Diagnosis

The rate of participants using the Liver Imaging Reporting and Data System (LI-RADS) as the imaging criteria for HCC was 62%, EASL 13%, and both 22%. While 38% never perform a biopsy for lesions with typical imaging characteristics, 24% perform a biopsy in LI-RADS 4 lesions, 18% always, and 8.9% when systemic treatment is planned. The approach for the hypovascular hepatobiliary phase hypointense lesions is decided according to LI-RADS by 41% of the participants, whereas 37% perform a biopsy, and 8.7% treat such lesions.

#### Consensus round: diagnosis

The majority (88.9%) of the participants recommend the LI-RADS reporting system for the diagnosis of HCC. For lesions with typical imaging characteristics, a biopsy is not mandatory for locoregional procedures in at-risk patients (89.2%); however, a biopsy is encouraged in patients without a prior histopathological evaluation (75.7%). In patients with HCC, the majority (73%) agreed that a histopathological analysis should be sought for additional hypovascular hepatobiliary phase hypointense lesions larger than 1 cm.

### Follow-up after thermal ablation

43% of the participants perform the first imaging after thermal ablation one month after the procedure, 21% after three months, and 17% immediately after the procedure. The majority (87%) repeat imaging every three months, and 83% prefer intensified follow-up for high-risk lesions (> 3 cm or > 3 lesions). The preferred imaging modality is MRI for 41%, both CT and MRI for 33%, and CT for 24%.

#### Consensus round: follow-up after thermal ablation

A consensus was not reached on the timing of the first follow-up after thermal ablation, neither for the first month (69.4%) nor for the first day (19.4%). However, participants reached a consensus that the imaging should be repeated every 3 months (91.7%) and that MRI should be preferred as the follow-up imaging modality (75.7%). No consensus was reached on additional CT imaging every 6 months (64.9%).

### Follow-up after TACE

The majority of participants (56%) perform the first follow-up imaging one month after TACE, and 93% of the participants repeat imaging every three months. 75% prefer intensified follow-up for high-risk lesions (large lesions/multiple nodules). The preferred imaging modality consisted of both CT and MRI for 50%, MRI for 32%, and CT for 16%.

#### Consensus round: follow-up after TACE

Consensus was reached that the timing of the first imaging should be within the first month after TACE (72.2%), and the interval for imaging should be every three months (94.6%). However, no consensus was reached that MRI should be the imaging modality for follow-up after TACE (63.9%), and that the follow-up has to be done with MRI after lipiodol-based TACE (69.4%).

### Follow-up after TARE

Forty-nine per cent of the participants scan patients undergoing TARE one month after the procedure, while 41% after three months. Most (89%) participants repeat imaging every three months, and 46% use both CT and MRI, while 32% use MRI and 19% use CT.

#### Consensus round: follow-up after TARE

Regarding follow-up after TARE, a consensus was reached that the third month should be the timing for the first imaging (86.5%), 3 months should be the imaging interval for follow-up (91.9%), and MRI should be the preferred imaging modality (72.2%).

### Follow-up after SBRT

While 59% of the participants were imaging patients after SBRT in the 3rd month, 41% were after one month. Most participants (87%) were repeating imaging every three months, while 55% were using CT and MRI, 29% MRI, and 13% CT for follow-up. 68% preferred intensified follow-up in high-risk lesions.

#### Consensus round: follow-up after SBRT

Participants reached a consensus that the timing of the first follow-up after SBRT should be the third month (97.3%), as well as that imaging should be repeated every three months (91.9%), and MRI should be the preferred imaging modality (77.1%).

### Response assessment

mRECIST (61%) was the most commonly used response assessment criterion, followed by LI-RADS (18%). 16% of the participants were using Response evaluation criteria in solid tumors (RECIST) criteria, and 4.1% the EASL criteria.

#### Consensus round: response assessment

The participants have reached a high consensus with 97.3% for the usage of perfusion-based imaging criteria (mRECIST, EASL, or LIRADS-TRA) for the response assessment after locoregional therapies.

### Artificial intelligence (AI)

Only 6.3% of the participants were using AI for treatment planning, while this ratio was 4.3% for usage of AI for the follow-up.

#### Consensus round: AI

A strong consensus was reached on the role of AI not being established in the treatment decision-making for locoregional therapies (97.3%), as well as follow-up after locoregional therapies (100%).

### Intraprocedural imaging

CT was the most commonly used imaging technique for guidance during thermal ablation (50%), followed by US (35%), fusion (8.7%), and MRI (6.5%). A majority (63%) prefer the alternative technique in case of poor visibility. The rate of participants performing cone beam CT during TACE and TARE was 55% and 61%, respectively. Half of the participants use CT for treatment planning in SBRT, while the rest use MRI.

#### Consensus round: intraprocedural imaging

All participants agreed that, depending on institutional experience, all imaging techniques (CT/US/MRI) can be used for guidance in thermal ablation. Similarly, the participants (85%) agreed that lipiodol TACE can be used prior to thermal ablation to improve visibility. Cone beam CT should be used to identify and confirm the vascular supply of lesions during TACE (88.6%) and TARE (94.3%). All participants agreed that CT or MRI can be used for treatment planning during SBRT.

The participant responses from the consensus (second) round are summarized in Table 1.

**Table 1:** Participant responses from the consensus round

Statement	Agreement	Consensus reached
Imaging work-up		
All patients should have CT and MRI to evaluate the local disease load in the liver.	83.8%	Yes
For the evaluation of local disease load in the liver, MRI should be obtained using gadoxetic acid.	89.2%	Yes
Abdomen CT for HCC patients should include arterial, portal, and venous phases.	97.3%	Yes
Abdomen CT for HCC patients should always include unenhanced images.	56.8%	No
Delay for the venous phase of Abdomen CT should be 180 s.	77.8%	Yes
DWI should always be obtained in the MRI of HCC patients.	89.2%	Yes
At the baseline, Thorax CT should always be obtained in all patients.	86.5%	Yes
PET-CT does not have to be part of the diagnostic work-up in HCC patients.	83.8%	Yes
If the liver imaging is older than one month before locoregional treatment, imaging should be repeated.	78.4%	Yes
The interval between imaging work-up for extrahepatic disease (Thorax CT) and locoregional treatment could be up to 3 months.	78.4%	Yes
Diagnosis		
LI-RADS reporting system should be used for the diagnosis of HCC.	88.9%	Yes
For the lesions with typical imaging characteristics, a biopsy does not have to be obtained for locoregional procedures.	89.2%	Yes
In cases with no prior histopathological evaluation, a biopsy is encouraged before locoregional therapies.	75.7%	Yes
In patients with HCC, histopathological analysis should be sought for additional hypovascular hepatobiliary phase hypointense lesions larger than 1 cm.	73%	Yes
Follow-up after thermal ablation		
The first imaging follow-up after thermal ablation should be performed within 1 month.	69.4%	No
The first imaging follow-up after thermal ablation should be performed within 1 day.	19.4%	No
Patients should be imaged for the liver every 3 months after thermal ablation.	91.7%	Yes
MRI should be preferred for follow-up imaging after thermal ablation.	75.7%	Yes
Every 6 months, CT imaging should be added for liver follow-up.	64.9%	No
Follow-up after TACE		
The first imaging follow-up after TACE should be performed within 1 month.	72.2%	Yes
Patients should be imaged every 3 months after TACE.	94.6%	Yes
MRI should be preferred for follow-up imaging after TACE.	63.9%	No
After lipiodol-based TACE, follow-up has to be done with MRI.	69.4%	No
Follow-up after TARE		
The first imaging follow-up after TARE should be performed within 3 months.	86.5%	Yes
Patients should be imaged every 3 months after TARE.	91.9%	Yes
MRI should be preferred for follow-up imaging after TARE.	72.2%	Yes
Follow-up after SBRT		
The first imaging follow-up after SBRT should be performed at 3rd month.	97.3%	Yes
Patients should be imaged every 3 months after SBRT.	91.9%	Yes
MRI should be preferred for follow-up imaging after SBRT.	77.1%	Yes
Response assessment		
Response assessment after locoregional therapies should use perfusion-based imaging criteria (mRECIST, EASL, or LIRADS-TRA).	97.3%	Yes
AI		
The role of AI has not been established in treatment decision-making for locoregional therapies.	97.3%	Yes
The role of AI has not been established in follow-up after locoregional therapies.	100%	Yes
Intraprocedural imaging		
Depending on institutional experience, all imaging techniques (CT/US/MRI) can be used for guidance in thermal ablation	100%	Yes
To improve visibility, lipiodol TACE can be used prior to thermal ablation.	85.%	Yes
Cone beam CT should be used to identify and confirm the vascular supply of lesions during TACE.	88.6%	Yes
Cone beam CT should be used to identify and confirm the vascular supply of lesions during TARE.	94.3%	Yes
Depending on institutional experience, CT or MRI can be used for treatment planning of SBRT.	100%	Yes

## Discussion

This paper offers expert recommendations on peri-interventional imaging for local and locoregional therapies in HCC, derived through a consensus survey utilizing the Delphi method. The first round of the survey identified the profile of the participants, as well as the existing variations between centers. During the Delphi process, participants reached a consensus for most points on the standardization of the periinterventional imaging.

Pretreatment imaging is needed to assess tumor burden within the liver, the presence of extrahepatic spread, the relationship of the tumor to surrounding structures, and to plan procedural details. However, the success of the pretreatment planning depends on the quality and timing of images. The participants agreed that all patients should undergo CT and MRI for the evaluation of the local disease load in the liver, and gadoxetic acid should be used as the contrast agent for MRI. MR imaging of the liver provides several advantages over CT, including superior soft-tissue resolution and tissue characterization, better delineation of the relationship with adjacent/central structures, and the ability to detect a greater number of lesions [4]; however, gadoxetic acid uptake by surrounding hepatocytes in the venous phase may be interpreted as wash-out of the lesion and decrease specificity [5, 6]. A retrospective analysis of over 30,000 HCC patients demonstrated that, among those with localized disease, individuals who underwent both CT and MRI with gadoxetic acid had significantly longer survival compared to those who underwent only CT or CT and non-gadoxetic acid-enhanced MRI [7]. The recommended abdomen CT protocol includes arterial, portal, and venous phase imaging, with a venous phase delay of 180 s. DWI is recommended as a mandatory sequence during MRI images, which is known to improve lesion detection and characterization [8]. Thorax CT was considered essential prior to locoregional therapies of HCC patients, whereas PET-CT was not included in the essential routine diagnostic work-up due to the low avidity of HCC lesions [9]. Considering the risk of tumor progression necessitating modification of treatment decision, repeating imaging was considered necessary if liver imaging was older than one month or thoracic imaging older than three months.

The LI-RADS reporting system is recommended for HCC diagnosis due to its high specificity and relevance for patients eligible for locoregional therapies, particularly those who may be future candidates for liver transplantation. LI-RADS provides standardization for HCC screening and surveillance with a detailed description of image acquisition, interpretation, and terminology [10]. Due to its high specificity, a biopsy is not deemed necessary if the lesion shows typical imaging characteristics. However, a biopsy is recommended if no prior histopathological evaluation exists, given the significance of tumor grade in prognostic prediction and to exclude the presence of mixed tumors or cholangiocarcinoma [11]. A biopsy is also recommended for hypovascular hepatobiliary phase hypointense lesions larger than 1 cm. While these lesions are not presently factored into treatment decision-making, the majority are classified as LI-RADS 4 and necessitate further diagnostic evaluation. Notably, a significant incidence of overt HCCs has been identified in these lesions, even those categorized as intermediate risk (LR-3) [12].

Imaging surveillance after local or locoregional therapies of the liver aims to assess anticipated changes in the treatment area, identify incomplete treatment, detect complications, recognize residual disease or local and distant recurrences, and predict survival, thereby informing subsequent therapeutic strategies. Although no consensus was reached for the timing of the first imaging after thermal ablation (ranging from the first day to 1st month), this was recommended as the 1st month for TACE and the 3rd month for TARE and SBRT. Repeating imaging every three months was suggested for all locoregional therapies. A consensus was reached that MRI should be preferred as the imaging modality for the follow-up after thermal ablation, TARE, and SBRT; for TACE, no consensus could be reached. Similarly, no consensus was reached for MRI being the imaging modality after lipiodol-based TACE. The prognostic value of lipiodol retention pattern in CT could be a reason for some participants to prefer CT for the follow-up [13]. However, this may also be attributable to the phrasing of the survey question (“has to be”), as suggested by the participants during the discussion following the second round. Considering there is currently no recommended treatment combining locoregional therapies with systemic agents in the guidelines, no recommendations were sought in this matter. However, additional research is needed to identify optimal imaging approaches if this situation changes with the ongoing trials.

Considering the distinct perfusion characteristics of HCC, participants recommended perfusion-based imaging criteria to assess response after locoregional therapies. However, due to the absence of clear superiority among the criteria, any of mRECIST, EASL, or LI-RADS-TRA (version 2018 at the time of the survey) was considered suitable for evaluating treatment response [5, 6, 14, 15]. With more evidence, a dedicated response assessment system for local therapies (such as LI-RADS-TRA version 2024) might be the first choice in the future.

Despite the growing number of studies assessing AI in HCC patients, the role of AI in the treatment decision-making process for locoregional procedures, as well as in post-treatment follow-up, has not yet been established and is not recommended due to the absence of randomized controlled trials.

The choice of intraprocedural imaging modality varies based on the institution, and all imaging modalities (US/CT/MRI) are deemed appropriate for lesion targeting in thermal ablation procedures. Each technique offers distinct advantages and limitations; therefore, imaging guidance should be selected based on the optimal visibility of the target, the center’s availability, and the operator´s expertise. Similarly, CT and MRI can be used for treatment guidance in SBRT. In addition to its additive tumoricidal effect, lipiodol-based TACE could improve lesion conspicuity, especially during CT- or CBCT-guided thermal ablation procedures [16]. Cone beam CT should be a part of the procedure to identify or confirm the vascular supply of lesions during TACE and TARE procedures, since superselective procedures are associated with better outcomes by increased tumor targeting and preservation of liver functions [17, 18].

This report has some limitations. Some recommendations are not supported by robust evidence from prospective studies and rely instead on expert opinion and consensus. However, this reflects the intent of our consensus: to fill existing data gaps and identify important questions that require investigation in future studies. Notably, two major European oncology and imaging societies were involved, and the survey included a wide range of specialists. Second, consensus could not be achieved on certain points despite a majority opinion, which may be partly related to the wording of some questions. Third, technical details for imaging and image reconstruction, such as slice thickness, reconstruction kernel, or dosage of contrast agents, were not addressed in this analysis.

In conclusion, this report outlines the recommendations of experts from oncological and radiological societies for the standardization of periinterventional imaging of HCC patients applicable to both daily clinical practice and prospective trials focused on local and locoregional therapies.

## Supplementary information

ELECTRONIC SUPPLEMENTARY MATERIAL

## Abbreviations

AI: Artificial intelligence
BCLC: Barcelona Clinic Liver Cancer
DWI: Diffusion-weighted images
EORTC: European Organisation for Research and Treatment of Cancer
ESGAR: European Society of Gastrointestinal and Abdominal Radiology
HCC: Hepatocellular carcinoma
LI-RADS: Liver imaging reporting and data system
SBRT: Stereotactic body radiation therapy
TACE: Transarterial chemoembolization
TARE: Transarterial radioembolization
