# Correction: Imaging in staging, treatment planning, and monitoring of hepatocellular carcinoma for local and locoregional therapies: consensus recommendations from EORTC and ESGAR

**DOI:** 10.1007/s00330-025-12045-7

**Published:** 2025-10-31

**Authors:** Osman Öcal, Christoph Johannes Zech, Maria Antonietta Bali, David Pasquier, Felix Mottaghy, Ingo Einspieler, Nikolaos Kartalis, Irene Bargellini, Roberto Iezzi, Timm Denecke, Wolfgang Gerhard Kunz, Bernhard Gebauer, Henning Wege, Roberto Cannella, Daniela Elena Oprea-Lager, Arndt Vogel, Bruno Sangro, Max Seidensticker

**Affiliations:** 1 Department of Diagnostic and Interventional Radiology, Heidelberg University Hospital, Heidelberg, Germany; 2 Imaging Group, European Organisation of Research and Treatment in Cancer (EORTC), Brussels, Belgium; 3 Radiology and Nuclear Medicine, University Hospital Basel, University of Basel, Basel, Switzerland; 4 European Society of Gastrointestinal and Abdominal Radiology (ESGAR), Vienna, Austria; 5 Radiology Department, Institut Jules Bordet Hôpital Universitaire de Bruxelles, Université Libre de Bruxelles, Brussels, Belgium; 6 Academic Department of Radiation Oncology, Centre Oscar Lambret, Lille, France; 7 CRIStAL, CNRS, Lille University, Lille, France; 8 Department of Radiology and Nuclear Medicine, Maastricht University Medical Center, Maastricht, The Netherlands; 9 Department of Nuclear Medicine, University Hospital RWTH Aachen University, Aachen, Germany; 10 Department of Radiology, University Hospital Regensburg, Regensburg, Germany; 11 Division of Radiology, Department of Clinical Science, Intervention and Technology (CLINTEC), Karolinska Institute, Stockholm, Sweden; 12 Diagnostic and Interventional Radiology Unit, Candiolo Cancer Institute, FPO-IRCCS, Candiolo, Italy; 13 Dipartimento di Diagnostica per Immagini e Radioterapia Oncologica, Fondazione Policlinico Universitario A. Gemelli IRCCS, UOC di Radiologia d’Urgenza e Interventistica, Roma, Italia; 14 Institute of Radiology, Catholic University, Rome, Italy; 15 Department of Diagnostic and Interventional Radiology, University of Leipzig, Leipzig, Germany; 16 Department of Radiology, University Hospital, LMU Munich, Munich, Germany; 17 Department of Radiology, Charité—University Medicine Berlin, Berlin, Germany; 18 Cancer Center Esslingen, Klinikum Esslingen, Esslingen, Germany; 19 Section of Radiology, Department of Biomedicine, Neuroscience and Advanced Diagnostics (BiND), University of Palermo, Palermo, Italy; 20 Department of Medical Imaging, Radboud University Medical Center, Nijmegen, The Netherlands; 21 Division of Gastroenterology and Hepatology, Toronto General Hospital, Toronto, ON, Canada; Medical Oncology, Princess Margaret Cancer Centre, Toronto, ON Canada; 22 Department of Gastroenterology, Hepatology, Infectious Diseases and Endocrinology, Hannover Medical School, Hannover, Germany; 23 Liver Unit and HPB Oncology Area, Clínica Universidad de Navarra and CIBEREHD, Pamplona, Spain; 24 Sapienza University of Rome, Rome, Italy; 25 La Fe Health Research Institute (IIS La Fe), Valencia, Spain; 26 Bern University Hospital (Inselspital), Bern, Switzerland; 27 Niels-Stensen-Kliniken Marienhospital, Osnabrück, Germany; 28 Amsterdam UMC, Amsterdam, The Netherlands; 29 Hacettepe University, Ankara, Turkey; 30 Ospedale del Mare, Naples, Italy; 31 Maastricht University Medical Center, Maastricht, The Netherlands; 32 UZ Leuven, Leuven, Belgium; 33 University and Hospital Trust, Verona, Italy; 34 Hampshire Hospitals NHS Foundation Trust, Basingstoke, UK; 35 University Hospital of Magdeburg, Magdeburg, Germany; 36 Leiden University Medical Center, Leiden, The Netherlands; 37 Ramón y Cajal University Hospital, Madrid, Spain; 38 University Hospitals Bristol, Bristol, UK; 39 Giresun University Hospital, Giresun, Turkey; 40 San Giovanni Hospital, Bellinzona, Switzerland; 41 University Hospital Regensburg, Regensburg, Germany; 42 Charité Universitätsmedizin Berlin, Berlin, Germany; 43 Department of Biomedical Sciences, Humanitas University, Pieve Emanuele, Milan, Italy; 44 Humanitas Cancer Center, IRCCS Humanitas Research Hospital, Rozzano, Milan, Italy; 45 Klinikum Würzburg Mitte, Würzburg, Germany

**Correction to: European Radiology**

10.1007/s00330-025-11699-7 published online 23 May 2025.

In the original article, affiliation of study group member Lorenza Rimassa’s affiliation was incorrectly given as IRCCS Humanitas Research Hospital, Roma, Italy. The correct affiliation is Humanitas Cancer Center, IRCCS Humanitas Research Hospital, Rozzano (Milan), Italy.

Lorenza Rimassa is also affiliated with Department of Biomedical Sciences, Humanitas University, Pieve Emanuele (Milan), Italy, which was left out in the original article.

Furthermore, the following Conflict of interest statement was missing in the original article:

L.R. has received consulting fees from AbbVie, AstraZeneca, Basilea, Bayer, BMS, Elevar Therapeutics, Exelixis, Genenta, Hengrui, Incyte, Ipsen, Jazz Pharmaceuticals, MSD, Nerviano Medical Sciences, Roche, Servier, Taiho Oncology, Zymeworks; lecture fees from AstraZeneca, Bayer, BMS, Eisai, Guerbet, Incyte, Ipsen, Roche, Servier; travel expenses from AstraZeneca, Servier; and institutional research funding from AbbVie, AstraZeneca, BeiGene, Exelixis, Fibrogen, Incyte, Ipsen, Jazz Pharmaceuticals, MSD, Nerviano Medical Sciences, Roche, Servier, Taiho Oncology, TransThera Sciences, Zymeworks.

The original article has been corrected.

