# Supplementary material for: Imaging in staging, treatment planning, and monitoring of hepatocellular carcinoma for local and locoregional therapies: consensus recommendations from EORTC and ESGAR
Source: Eur Radiol. 2025 May 23;36(1):597–606. doi: 10.1007/s00330-025-11699-7 (PMC12712117; doi:10.1007/s00330-025-11699-7)
Supplement: Supplementary file 1 — ELECTRONIC SUPPLEMENTARY MATERIAL [file 330_2025_11699_MOESM1_ESM.pdf]

Imaging in staging, treatment planning, and monitoring of hepatocellular carcinoma for local and locoregional therapies: Consensus recommendations from EORTC and ESGAR

ELECTRONIC SUPPLEMENTARY MATERIAL

Eur Radiol (2025) Öcal O, Zech CJ, Bali MA, et al.

Round 1

* Indicates required question

1. Email *

2. Name

Institutional details

3. 1. Which center do you work at? *

4. In which country do you work? *

5. 2. What is your profession? *

Mark only one oval.

- General imaging
- Nuclear imaging
- Interventional radiologist
- Medical oncologist
- Hepatologist/Gastroenterologist
- Radiation oncologist
- Surgeon
- Other:

6. 3. How many thermal ablations (for HCC) were performed yearly in your institution in the last five years?

7. 4. How many TACE (for HCC) were performed yearly in your institution in the last five years?

8. 5. How many TARE (for HCC) were performed yearly in your institution in the last five years?

9. 6. How many SBRT (for HCC) were performed yearly in your institution in the last five years?

Preprocedural imaging

10. 7. Which imaging do you prefer for treatment planning in HCC patients? *

Mark only one oval.

- CT
- MRI
- Both CT and MRI
- PET/CT
- Other:

11. 8. In case you use MRI for planning, how often do you use gadoxetic acid?

Mark only one oval.

- Never
- Always
- Other:

12. 9. Which CT protocol is used in your institution for HCC patients?

Mark only one oval.

- Arterial + Portal
- Arterial + Portal + Venous
- Unenhanced + Arterial + Portal + Venous
- Other:

13. 10. Which delay do you prefer for venous phase in CT?

Mark only one oval.

- 120 seconds
- 180 seconds
- Other:

14. 11. Do you routinely obtain diffusion-weighted imaging in MRI for HCC patients?

Mark only one oval.

- Yes
- No

15. 12. How often do you obtain Thorax-CT in HCC patients?

Mark only one oval.

- Never
- Always
- Other:

16. 13. How often do you obtain PET-CT in HCC patients?

Mark only one oval.

- Never
- Always
- Other:

17. 14. How long interval between imaging and procedure do you accept for liver imaging? *

Mark only one oval.

- Up to 1 week
- Up to 1 month
- Up to 3 months
- Other:

18. 15. How long interval between imaging and procedure do you accept for extrahepatic imaging? *

Mark only one oval.

- Up to 1 week
- Up to 1 month
- Up to 3 months
- Other:

19. 16. Which imaging criteria do you (your department) use in reports for HCC diagnosis?

Mark only one oval.

- EASL
- LI-RADS
- Both
- Other:

20. 17. In lesions with typical imaging characteristics, how often do you perform a biopsy?

Mark only one oval.

- Never
- In LI-RADS 4 lesions
- In patients listed for transplantation
- Always
- Other:

21. 18. What is your approach for hypovascular hepatobiliary phase hypointense lesions?

Mark only one oval.

- No additional approach
- Watch and wait
- According to LI-RADS
- Biopsy
- Treatment (Ablation or recommended treatment according to the BCLC scheme)

Treatment preferences

22. 19. Which treatment is preferred in your institution for patients with BCLC-0?

Mark only one oval.

- Ablation
- Resection
- TACE/TARE (if not amenable for resection or ablation)
- SBRT
- Other:

23. 20. Which treatment is preferred in your institution for patients with BCLC-A?

Mark only one oval.

- Ablation
- Resection
- TACE/TARE (if not amenable for resection or ablation)
- SBRT
- Other:

24. 21. Which treatment is used primarily as bridging in patients listed for transplantation?

Mark only one oval.

- Ablation
- TACE
- TARE
- TACE+Ablation
- SBRT
- Other:

25. 22. Which treatment is used primarily for downstaging in patients listed for transplantation?

Mark only one oval.

- Ablation
- TACE
- TARE
- TACE+Ablation
- SBRT
- Other:

26. 23. Do you screen patients under systemic treatment for potential locoregional treatments?

Mark only one oval.

- Yes
- No
- Other:

27. 24. In which cases do you combine ablation and TACE in BCLC 0 and A patients?

Mark only one oval.

- Always
- >3cm
- >5cm
- Difficult locations
- Never
- Other:

28. 25. In which conditions do you avoid thermal ablation?

Tick all that apply.

- Never
- Proximity to liver hilum
- Subcapsular location
- Perivascular location
- Other:

29. 26. In which conditions do you avoid TACE?

Tick all that apply.

- Never
- Hypovascular lesions
- >5cm
- According to some criteria
- Other:

30. 27. In which conditions do you avoid TARE?

Tick all that apply.

- Never
- Hypovascular lesions
- >8cm
- According to some criteria
- Other:

31. 28. In which conditions do you avoid SBRT?

Tick all that apply.

- Never
- Proximity to liver hilum
- Subcapsular location
- Perivascular location
- Other:

Intraprocedural imaging

32. 29. Which imaging technique do you prefer for needle placement in thermal ablation?

Mark only one oval.

- Ultrasound
- CT
- MRI
- Fusion

33. 30. In which conditions do you choose an alternative imaging technique?

Tick all that apply.

- Small lesions
- Poor visibility
- Other:

34. 31. How often do you obtain Dyna-CT in TACE?

Mark only one oval.

- Never
- Always
- Other:

35. 32. How often do you obtain Dyna-CT in TARE?

Mark only one oval.

- Never
- Always
- Other:

36. 33. Which imaging technique do you prefer for SBRT?

Mark only one oval.

- CT
- MRI
- Other:

Postprocedural imaging-thermal ablation

37. 34. When do you image patients for the first time after thermal ablation?

Mark only one oval.

- Immediately after the procedure
- Next day
- 1 month
- 3 months
- Other:

38. 35. How often do you image patients after thermal ablation?

Mark only one oval.

- Every month
- Every 2 months
- Every 3 months
- Every 6 months
- Other:

39. 36. In which conditions do you prefer intensified follow-up after local ablation?

Mark only one oval.

- High-risk lesions (>3 cm lesions or more than 3 lesions)
- High risk patients (active viral hepatitis or alcoholic etiology)
- Other:

40. 37. Which imaging method do you use for follow-up after thermal ablation?

Mark only one oval.

- Ultrasound
- CT
- MRI
- Both CT and MRI
- PET/CT
- Other:

Postprocedural imaging

41. 38. When do you image patients for the first time after TACE?

Mark only one oval.

- Immediately after the procedure
- Next day
- 1 month
- 3 months
- Other:

42. 39. How often do you image patients after TACE?

Mark only one oval.

- Every month
- Every 2 months
- Every 3 months
- Every 6 months
- Other:

43. 40. In which conditions do you prefer intensified follow-up after TACE?

Tick all that apply.

- High-risk lesions (large lesions/multiple nodules)
- High risk patients (active viral hepatitis or alcoholic etiology)
- Other:

44. 41. Which imaging method do you use for follow-up after TACE?

Mark only one oval.

- Ultrasound
- CT
- MRI
- Both CT and MRI
- PET/CT
- Other:

Postprocedural imaging

45. 42. When do you image patients for the first time after TARE?

Mark only one oval.

- Immediately after the procedure
- Next day
- 1 month
- 3 months
- Other:

46. 43. How often do you image patients after TARE?

Mark only one oval.

- Every month
- Every 2 months
- Every 3 months
- Every 6 months
- Other:

47. 44. Which imaging method do you use for follow-up for TARE?

Mark only one oval.

- Ultrasound
- CT
- MRI
- Both CT and MRI
- PET/CT
- Other:

Postprocedural imaging-SBRT

48. 45. When do you image patients for the first time after SBRT?

Mark only one oval.

- Immediately after the procedure
- Next day
- 1 month
- 3 months
- Other:

49. 46. How often do you image patients after SBRT?

Mark only one oval.

- Every month
- Every 2 months
- Every 3 months
- Every 6 months
- Other:

50. 47. In which conditions do you prefer intensified follow-up after SBRT?

Tick all that apply.

- High-risk lesions
- High-risk patients
- Other:

51. 48. Which imaging method do you use for follow-up after SBRT?

Mark only one oval.

- Ultrasound
- CT
- MRI
- Both CT and MRI
- PET/CT
- Other:

Response evaluation

52. 49. Which response criteria do you use for response evaluation?

Mark only one oval.

- RECIST
- mRECIST
- EASL
- LI-RADS
- Other:

Artificial intelligence

53. 50. Do you use AI for treatment planning?

Mark only one oval.

- Yes
- No

54. 51. Do you use AI for follow-up?

Mark only one oval.

- Yes
- No

This content is neither created nor endorsed by Google.

Round 2

* Indicates required question

1. Your email address: *

Imaging work-up

2. 1. All patients should have CT and MRI to evaluate the local disease load in the liver.

Mark only one oval.

( ) Yes
( ) No

3. 2. For the evaluation of local disease load in the liver, MRI should be obtained using gadoxetic acid.

Mark only one oval.

( ) Yes
( ) No

4. 3. Abdomen CT for HCC patients should include arterial, portal, and venous phases.

Mark only one oval.

( ) Yes
( ) No

5. 4. Abdomen CT for HCC patients should always include unenhanced images.

Mark only one oval.

( ) Yes
( ) No

6. 5. Delay for the venous phase of Abdomen CT should be 180 seconds.

Mark only one oval.

( ) Yes
( ) No

7. 6. Diffusion-weighted images should always be obtained in MRI of HCC patients.

Mark only one oval.

( ) Yes
( ) No

8. 7. At the baseline, Thorax CT should always be obtained in all patients.

Mark only one oval.

( ) Yes
( ) No

9. 8. PET-CT does not have to be part of the diagnostic work-up in HCC patients.

Mark only one oval.

( ) Yes
( ) No

10. 9. If liver imaging is older than one month before locoregional treatment, imaging should be repeated.

Mark only one oval.

( ) Yes
( ) No

11. 10. The interval between imaging work-up for extrahepatic disease (Thorax CT) and locoregional procedure could be up to 3 months.

Mark only one oval.

( ) Yes
( ) No

Diagnosis

12. 11. LI-RADS reporting system should be used for the diagnosis of HCC.

Mark only one oval.

( ) Yes
( ) No

13. 12. For lesions with typical imaging characteristics, a biopsy does not have to be obtained for locoregional procedures.

Mark only one oval.

( ) Yes
( ) No

14. 13. In cases with no prior histopathological evaluation, a biopsy is encouraged before locoregional therapies.

Mark only one oval.

( ) Yes
( ) No

15. 14. In patients with HCC, histopathological analysis should be sought for additional hypovascular hepatobiliary phase hypointense lesions larger than 1cm.

Mark only one oval.

( ) Yes
( ) No

Follow-up

Follow-up after thermal ablation

16. 15. The first imaging follow-up after thermal ablation should be performed within 1 month.

Mark only one oval.

( ) Yes
( ) No

17. 16. The first imaging follow-up after thermal ablation should be performed within 1 day.

Mark only one oval.

( ) Yes
( ) No

18. 17. Patients should be imaged for liver every 3 months after thermal ablation.

Mark only one oval.

( ) Yes
( ) No

19. 18. MRI should be preferred for follow-up imaging modality after thermal ablation.

Mark only one oval.

( ) Yes
( ) No

20. 19. Every 6 months, CT imaging should be added for liver follow-up.

Mark only one oval.

( ) Yes
( ) No

Follow-up after TACE

21. 20. The first imaging follow-up after TACE should be performed within 1 month.

Mark only one oval.

( ) Yes
( ) No

22. 21. Patients should be imaged every 3 months after TACE.

Mark only one oval.

( ) Yes
( ) No

23. 22. MRI should be preferred for follow-up imaging modality after TACE.

Mark only one oval.

( ) Yes
( ) No

24. 23. After lipiodol-based TACE, follow-up has to be done with MRI.

Mark only one oval.

( ) Yes
( ) No

Follow-up after radioembolization

25. 24. The first imaging follow-up after TARE should be performed within 3 months.

Mark only one oval.

( ) Yes
( ) No

26. 25. Patients should be imaged every 3 months after TARE.

Mark only one oval.

( ) Yes
( ) No

27. 26. MRI should be preferred for follow-up imaging modality after TARE.

Mark only one oval.

( ) Yes
( ) No

Follow-up after SBRT

28. 27. The first imaging follow-up after SBRT should be performed at 3rd month.

Mark only one oval.

( ) Yes
( ) No

29. 28. Patients should be imaged every 3 months after SBRT.

Mark only one oval.

( ) Yes
( ) No

Response assessment

30. 29. MRI should be preferred for follow-up imaging modality after SBRT.

Mark only one oval.

( ) Yes
( ) No

31. 30. Response assessment after locoregional therapies should use perfusion-based imaging criteria (mRECIST, EASL, or LIRADS-TRA).

Mark only one oval.

( ) Yes
( ) No

Artificial intelligence

32. 31. The role of artificial intelligence has not been established in treatment decision-making for locoregional therapies.

Mark only one oval.

( ) Yes
( ) No

33. 32. The role of artificial intelligence has not been established in follow-up after locoregional therapies.

Mark only one oval.

( ) Option 1

Intraprocedural imaging

34. 33. Depending on institutional experience, all imaging techniques (CT/US/MRI) can be used for guidance in thermal ablation.

Mark only one oval.

( ) Yes
( ) No

35. 34. To improve visibility, lipiodol TACE can be used prior to thermal ablation.

Mark only one oval.

( ) Yes
( ) No

36. 35. Cone beam CT should be used to identify or confirm the vascular supply of lesions during TACE.

Mark only one oval.

( ) Yes
( ) No

37. 36. Cone beam CT should be used to identify or confirm the vascular supply of lesions during TARE.

Mark only one oval.

( ) Yes
( ) No

38. 37. Depending on institutional experience, CT or MRI can be used for treatment planning of SBRT.

Mark only one oval.

( ) Yes
( ) No

This content is neither created nor endorsed by Google.
